# Understanding the Impact of Industrial Stress Conditions on Replicative Aging in *Saccharomyces cerevisiae*

**DOI:** 10.3389/ffunb.2021.665490

**Published:** 2021-06-02

**Authors:** Marco Eigenfeld, Roland Kerpes, Thomas Becker

**Affiliations:** Research Group Beverage and Cereal Biotechnology, Institute of Brewing and Beverage Technology, Technical University of Munich, Freising, Germany

**Keywords:** yeast, stress response, cell age, replicative aging, aging, age distribution

## Abstract

In yeast, aging is widely understood as the decline of physiological function and the decreasing ability to adapt to environmental changes. *Saccharomyces cerevisiae* has become an important model organism for the investigation of these processes. Yeast is used in industrial processes (beer and wine production), and several stress conditions can influence its intracellular aging processes. The aim of this review is to summarize the current knowledge on applied stress conditions, such as osmotic pressure, primary metabolites (e.g., ethanol), low pH, oxidative stress, heat on aging indicators, age-related physiological changes, and yeast longevity. There is clear evidence that yeast cells are exposed to many stressors influencing viability and vitality, leading to an age-related shift in age distribution. Currently, there is a lack of rapid, non-invasive methods allowing the investigation of aspects of yeast aging in real time on a single-cell basis using the high-throughput approach. Methods such as micromanipulation, centrifugal elutriator, or biotinylation do not provide real-time information on age distributions in industrial processes. In contrast, innovative approaches, such as non-invasive fluorescence coupled flow cytometry intended for high-throughput measurements, could be promising for determining the replicative age of yeast cells in fermentation and its impact on industrial stress conditions.

## Introduction

Yeasts are indispensable organisms in various industrial processes, such as wine, cider, and beer making and, more recently, in biofuel production. Many single-cell organisms propagate by symmetrical splitting into two virtually identical entities that do not age and are, therefore, considered potentially immortal. While these cells can die due to non-age-related causes, such as disease or injury, they do not die due to senescence (Petralia et al., 2014). The life cycle of budding yeast differs from this process, as it does not propagate by symmetrical cell division. Hence, there is a need to define aging in yeast cells.

**Figure 1:**
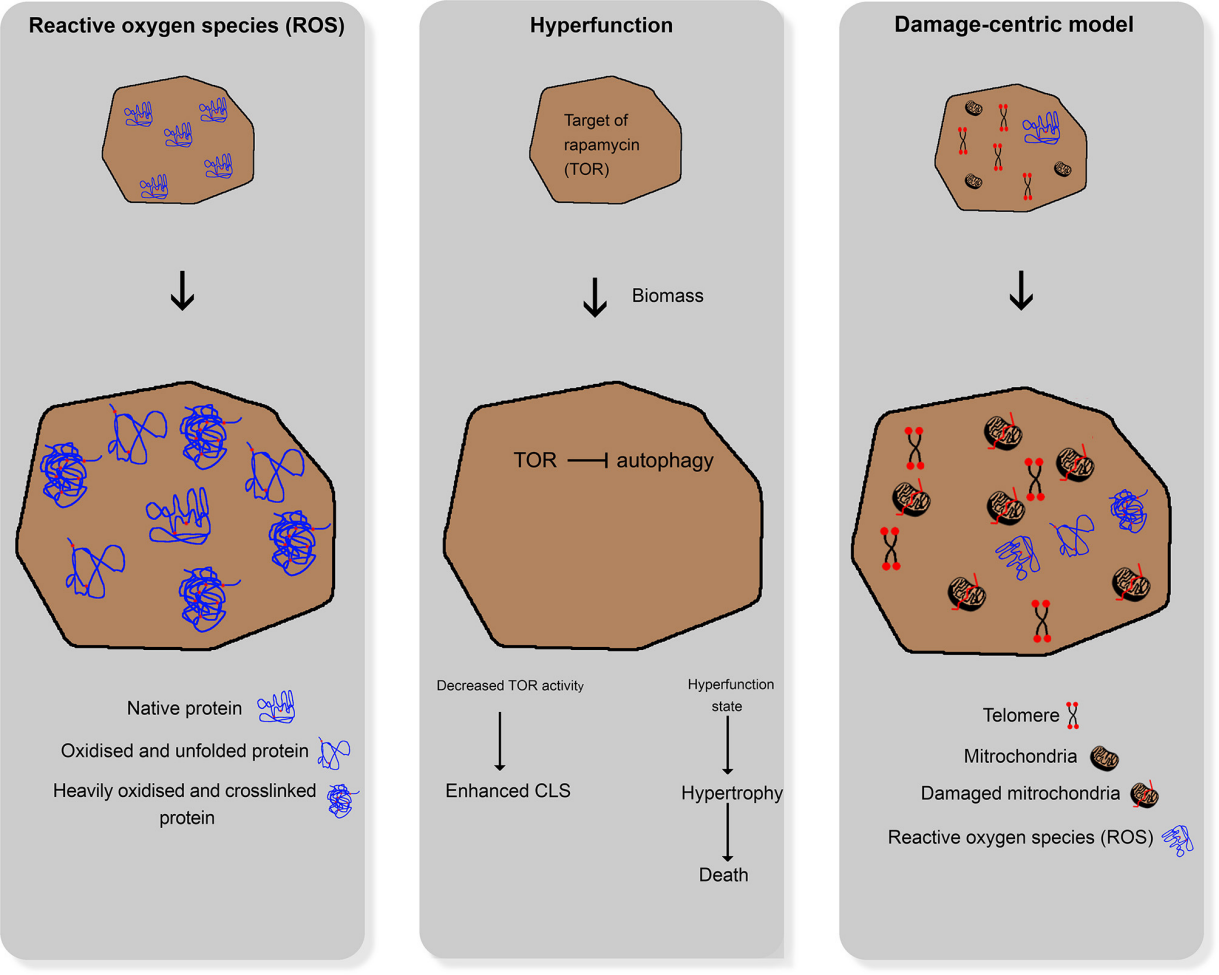
Comparative illustration of the three main aging theories for higher organisms; left: reactive oxygen species damaging cellular structures; middle: hyperfunction theory; right: damage-centric model.

In general, aging is defined as the ability of an organism to adapt to environmental changes (Martin and Hofer, 2004). Aging and age-associated physiological processes have been intensively investigated in numerous scientific studies in recent years (Leupold et al., 2019; Chen et al., 2020; Kim and Benayoun, 2020). Given this fact, three major theories, namely the reactive oxygen species theory, hyperfunction theory, and damage-centric model, have evolved (Figure 1). These theories deal with the characterization and explanation of aging. Relevant questions include how aging can be observed within different populations and between other species, as well as its psychological, social, and physiological aspects. Not all of these theories are transferable to the aging of yeast cells. Moreover, daughter yeast cells are theoretically immortal as long as the environmental stress does not influence homeostasis.

In industrial processes, the yeast cultures face multiple parallel factors that may decrease cell viability and result in the aging of yeast cells due to disrupted homeostasis. Current methods for investigating cell aging in yeast cultures are mainly based on time-consuming processes or cell separation, but not on yeast culture fractionation, for the measurement of distributions of yeast cell ages in whole populations.

In the past years, many reviews were published focusing on the impact of telomere length (Liu et al., 2019; Harari et al., 2020), oxidative stress (Eleutherio et al., 2018), DNA and cell size (Veitia, 2019), and genetics (He et al., 2018; Lee and Ong, 2021), metabolic pathways (Leupold et al., 2019) to replicative aging and cellular senescence of budding yeast. Furthermore, only a few reviews focus on models for the analysis of aging (Denoth Lippuner et al., 2014; O'Laughlin et al., 2020). However, there is a lack of publications focusing on the impact of stress on replicative aging and fast determination methods, analyzing the replicative age distribution in populations.

This review focuses on the interconnection of physiological changes and aging of yeast populations, including the impact of industrial relevant stressors on replicative aging and their determination.

## General Aspects Of Aging

### Differences in Aging Theories

In the past years, different approaches evolved to explain aging in humans. One approach assumes that reactive oxygen species (ROS) and reactive nitrogen species (RNS), which are formed during several metabolic processes under aerobic conditions, damage cellular structures (e.g., DNA and proteins). These ROS and RNS accumulate over time, leading to loss of physiological cellular, tissue, and organ function. Hence, they can be used as markers of aging (Beckman and Ames, 1998). Cells can defend themselves against ROS and RNS by enzymatic pathways, such as the glutathione peroxidase pathway, superoxide dismutase, and catalase, and non-enzymatic endogenous antioxidants, such as vitamin C (ascorbic acid), vitamin E (α-tocopherol), bilirubin, and uric acid (Liguori et al., 2018). Furthermore, there is strong evidence that reactive oxygen and nitrogen species (RONS) and oxidative stress lead to cellular senescence with a characteristic and irreversible senescence-associated secretory phenotype (Pole et al., 2016).

The hyperfunction theory states that the cellular programs necessary for development and growth continue to work after completion of the growth phase, thereby driving senescence as a run-on of cellular growth (Gems and de la Guardia, 2013). Interestingly, substances (e.g., rapamycin and metformin) that target the associated cellular pathways can retard aging in cell cultures (Blagosklonny, 2012). According to this theory, the aging process in yeast cells leads to significant changes and defects at various levels that reflect the adaptation of yeast cells to multiple kinds of stressors.

The third theory of aging focuses on damages induced by the reaction of cells to stressors. It states that, in the course of life, a multitude of smaller cellular damages is acquired and accumulated in the organism, eventually leading to the collapse of the organism and its death. This theory relies on molecular biological changes that can be observed in aging organisms (Zimniak, 2012), e.g., oxidative (Koc et al., 2004), and genetic (Failla, 1958; Partridge, 2007) damages. Thus, native cultures of mammalian cells eventually become proliferative and enter a state of senescence. Moreover, the number of senescent cells in older organisms increases in all tissues compared with younger ones (Baker et al., 2011). Models of accumulation of acquired damage over time can be described as damage-centric models that focus on particular types of damage (Kirkwood and Austad, 2000).

Not all of these theories are transferable to the aging of yeast cells in industrial processes. As shown above, a definition of the general lifespan from birth until death is not suitable for yeast. Hence, the age of yeast cells is not only aligned to the course from budding to death (chronological lifespan); instead, it additionally involves a number of cell divisions until the onset of senescence of the mother cell (replicative lifespan) (Mortimer and Johnston, 1959; Longo et al., 2012). The following section focuses on aspects of the third theory, which deals with the characteristics of acquired and accumulating molecular changes within the organism. Yeast has become an important model organism in aging research, and aging-specific aspects are discussed below (Breitenbach et al., 2011).

### Mechanisms of Aging

Various mechanisms of aging are being investigated to explain aging and aging-related processes in yeast as well as higher organisms. These include an alternation of telomeres with age and an increase in oxidative stress, which eventually lead to molecular damage, epigenetic regulation, genomic stability, and DNA repair linked to oxidative stress. These processes are explained in the following sections in more detail.

#### Telomeres and Telomerase Complex

The role of telomeres and the telomere-synthesizing enzyme complex telomerase has attracted attention early during the investigation of aging and age-related processes (D'Mello and Jazwinski, 1991; Blackburn et al., 2006) in yeast. With each replication, the ends of the chromosomes shorten by ~25–200 bp. Therefore, telomeres appear to determine the maximum number of cell divisions and can be referred to as “mitotic clock” (Allsopp et al., 1992). Together with specialized proteins, telomeres comprise the ends of linear chromosomes that exist as repetitive non-protein coding tandems of 300 ± 75 bp (Wellinger and Zakian, 2012), protecting the chromosomes against sequence loss due to incomplete DNA replication (Bertuch and Lundblad, 1998) and consequent shortening. In this way, telomeres and telomere proteins play a crucial role in protecting DNA from degradation or fusion events; thus, they serve genomic integrity (Bianchi and Shore, 2008; Wellinger, 2009). Since telomeres are shortened with each cell division cycle, they eventually reach a critical length with age (Harley et al., 1990; Jay et al., 2016), at which cellular senescence is initiated; this state is known as replicative senescence (Barrientos-Moreno et al., 2018). Through a genomic damage signal cascade, apoptosis is finally induced (Hayflick and Moorhead, 1961; Allsopp et al., 1992; Maser and DePinho, 2004; Rice and Skordalakes, 2016). The telomerase complex can influence these telomeres to control their length (Greider and Blackburn, 1985, 1987). It consists of the telomerase reverse transcriptase of telomerase RNA (TR), which serves as a template for synthesis (Greider and Blackburn, 1989; Feng et al., 1995; Lingner et al., 1997). Damage due to inactivation of telomerase results in a DNA damage checkpoint response, which leads to changes in size, morphology, and cellular senescence in *Saccharomyces cerevisiae* (*S. cerevisiae*) cells after ~60–70 cell cycles (Ghanem et al., 2019).

#### Endogenous Oxidative Stress

Another contributor to age-acquired molecular damage is a state referred to as oxidative stress. This state is the result of RONS (see section Differences in Aging Theories) produced during normal cellular metabolism (Ceriello et al., 2016) and their accumulation over time, which damages the intracellular enzymes. Endogenous sources of such reactive species include the enzymatic activity of nicotinamide adenine dinucleotide phosphate oxidase (Salisbury and Bronas, 2015). The resulting enzymatic product leads to the conversion of the radical superoxide anion to H_2_O_2_ by superoxide dismutase.

RONS affect the intracellular components, including the lipids of plasma membranes, proteins, carbohydrates (Kaludercic and Giorgio, 2016) negatively. RONS also impair the DNA molecules, resulting in mutations, and cytotoxicity (Kay et al., 2019).

To avoid such cell damage, dysfunction, and death (Kaludercic and Giorgio, 2016), RONS are rapidly removed via enzymatic degradation. Some of the common examples of RONS include superoxide, alkoxyl, hydrochlorous acid, hydrogen peroxide (H_2_O_2_), nitric acid, peroxynitrite, and nitrogen dioxide (Li et al., 2016; Möller et al., 2019).

#### Epigenetic Regulation

Closely linked to the effects of oxidative stress on senescence-associated secretory phenotype is the question of the genomic integrity of a cell. Generally, genomic stability is considered an essential factor in cellular aging (López-Otín et al., 2013). Hence, the maintenance of genomic integrity is regarded as an important factor in describing the process of aging in various species. DNA damage is crucial for genomic stability (e.g., mediated through RONS). As mentioned above, RONS are largely produced during normal cellular metabolism, and some authors believe that RONS cause >10,000 DNA lesions daily (Lindahl, 1993; Martin, 2008; Cadet and Wagner, 2013). As with oxidative stress, eukaryotic cells have a large set of defense mechanisms to neutralize the effects of DNA damage and a DNA repair mechanism. In unicellular and higher organisms, these defense mechanisms are crucial for the prevention of premature aging and degenerative diseases, cellular homeostasis, and pre- and post-natal development (Abbas et al., 2013; Vijg and Suh, 2013; Maynard et al., 2015). These mechanisms can repair a variety of DNA lesions, e.g., single- and double-strand breaks, cross-links, and base-pair mismatches (Ciccia and Elledge, 2010; O'Driscoll, 2012; Chong et al., 2019).

The relationship between genomic integrity and health can be shown through genomic instability syndromes, which affect one or more DNA damage repair mechanisms. For example, heterozygosity for mutations in specific mismatch repair proteins p53 and BRCA1/2 (Roy et al., 2011; Peña-Diaz and Jiricny, 2012; Sorrell et al., 2013; Williams and Schumacher, 2016) can be associated with chromosomal instabilities, predisposition to cancer, degeneration of particular tissues, or hypersensitivity to DNA-damaging agents (O'Driscoll, 2012; Maynard et al., 2015). Nevertheless, besides cancer, cardiovascular or metabolic diseases may also develop as a result of the loss of such DNA damage repair mechanisms (Shimizu et al., 2014).

Besides telomeres and oxidative damage, also epigenetic regulation mechanisms appear to be involved in the individual aging process of an organism. One epigenetic aspect crucial for the aging process appears to be the expression of core histones, which are critical to chromatin structure, and linked to DNA replication and repair (Cavalli and Misteli, 2013). In yeast, the expression of core histones is decreased during replicative aging. A reduction in histone expression is associated with reduced nucleosome packaging density and, hence, upregulation of associated genes (Hu et al., 2014). Furthermore, it has been shown that complexes control histone expression, as well as the exchange and deposition on the chromatin, positively, and negatively influence the replicative lifespan of yeast (Feser et al., 2010).

In addition, it was shown that the methylation status of CpG dinucleotides (classically linked to transcriptional silencing) varies in senescent and actively cycling human cell cultures (Wilson and Jones, 1983; Cruickshanks et al., 2013). In contrast, such an epigenetic modification does not exist in aging yeast cells (Capuano et al., 2014). Although the absence of DNA methylation in several yeast strains remains unclear, it could be assumed that yeast lineages lost this epigenetic control mechanism early in evolution (Zemach et al., 2010).

As shown in the sections above, there is likely no singular aging mechanism in higher species, such as yeasts. Thus, aging may be understood as the synergistic result of several physiological processes, changes, damages, and repair events. Aging has extensively been studied in the *S. cerevisiae*, the budding yeast, and significant findings will be discussed in this review.

### Aging in Yeast

Alternatively, aging is theorized as a programmed cellular process that can be accelerated or retarded, but not arrested. Cellular growth path, such as the target of rapamycin, a regulatory path of growth and protein translation (Kennedy and Lamming, 2016), have been implicated in controlling this programmed, chronological aging. In replicative aging, the number of divisions a cell undergoes is considered a molecular clock. It allows a given cell to undergo a fixed number of cell divisions before it dies (Mortimer and Johnston, 1959; Longo et al., 2012). This model is true for mammalian cells in tissue culture, but applies to yeast cells only when modified.

**Figure 2:**
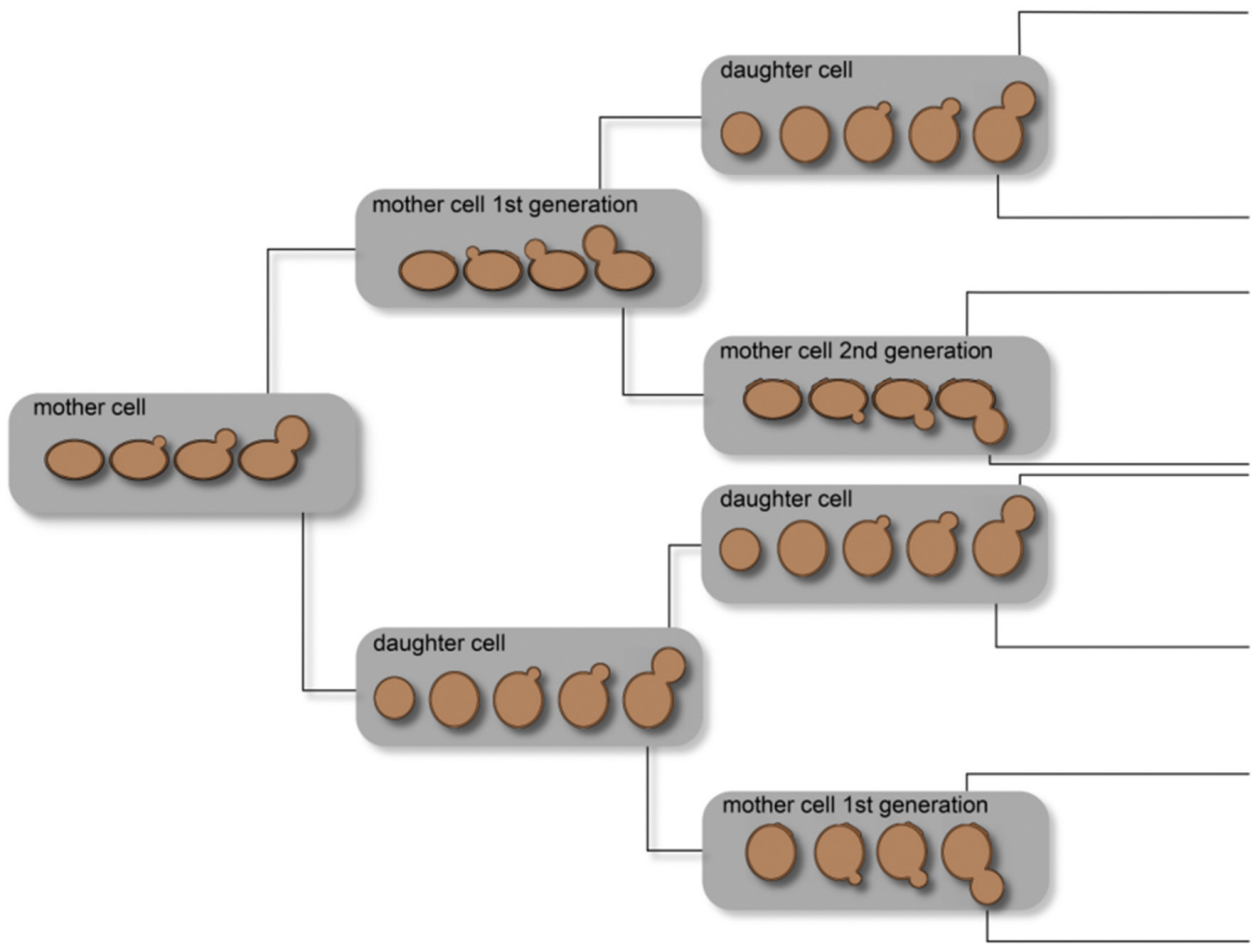
Illustration of the age distribution in yeast cell populations. Owing to the budding process of a mother cell, a daughter cell is created and the mother cell results in a mother cell of the subsequent generation. Thus, a cell culture theoretically consists of 50% daughter cells, 25% mother cells of the first generation, 12.5% of the second generation, and so on.

In yeast, aging is influenced by several simultaneous events, such as (1) telomeres and telomere replication protein complex, (2) endogenous oxidative stress, and (3) epigenetic regulation mechanisms. These mechanisms have impact on the chronological and replicative aging of yeast cells. One of the first experiments to study the replicative lifespan of individual yeast cells was described by Mortimer and Johnston (1959). Through microdissection, explained in section Micromanipulation Techniques, they revealed that the number of divisions is limited in yeast. Bud scars, accumulating on the surface of the mother cell where the bud developed, distinguish mother and daughter cells. It was described that, at the side of the scar, there is no development of new buds; therefore, the non-scarred cell surface at least limits the lifespan of yeast cells. Starting with 36 mother cells, the observed mean lifespan was 23.9 generations, with a standard deviation of 8.3 generations. This experiment was the first to show that yeast cells are mortal. Depending on the specific strain, the lifespan varies between 25 and 35 budding events (Johnston, 1966; Müller, 1971). In addition, the replicative lifespan is influenced by the conditions under which the yeast cultures are maintained. Figure 2 illustrates single-cell lineages and the classification of cell types into mother and daughter cells under ideal conditions. This demonstrates a non-normal distribution of the yeast cell age in whole populations influenced by the molecular mechanisms occurring in every single cell.

It is known, that in yeast, the corresponding tandem sequence varies within the genus *Saccharomyces* (Cohn et al., 1998). It consists of double-stranded G-rich base-pair arrays of irregular repeats (McEachern and Blackburn, 1994), such as (TG)_1−4_G_2−3_ in *S. cerevisiae* (Cohn et al., 1998; Förstemann and Lingner, 2001), and 12–16 G-bases at the 3′ end as a G-overhang (Wellinger et al., 1993). This sequence is highly conserved in yeast by a unique protein complex termed Cdc13p–Stn1p–Ten1p. This complex plays an essential role in providing chromosome end protection and maintaining telomere homeostasis. Cdc13p is the main protein of this homeostatic mechanism, binding to single-strand DNA and regulating telomere replication (Pennock et al., 2001). It also associates with Stn1p and Ten1p to assemble the Cdc13p–Stn1p–Ten1p complex for telomere protection (Ge et al., 2020). This complex additionally ensures that the ends of a chromosome are not inadvertently recognized as a DNA strand break by the cellular DNA repair mechanisms. Interestingly, overlengthened telomeres have no impact on yeast chronological life span (Harari et al., 2017). It could also be shown that the exposure of yeast to low concentrations of ethanol results in a high telomere elongation (Romano et al., 2013).

Another contributor to cellular stress and a variation in replicative lifespan is oxidative stress. In 2001, Davidson and Schiestl demonstrated that lethal heat stress (exposure to 50°C) is also associated with elevated endogenous oxidative stress, possibly resulting in an increased nuclear mutation frequency (Davidson and Schiestl, 2001). The authors assumed the involvement of the mitochondrial respiratory electron carriers. Therefore, they investigated the heat vulnerability of coenzyme Q- and nicotinamide adenine diphosphate dehydrogenase-deficient yeast strains. They also demonstrated that cells lacking coenzyme Q secreted up to 30-fold higher levels of H_2_O_2_ at 42°C than at 30°C. In contrast, nicotinamide adenine diphosphate dehydrogenase-deficient cells did not secrete H_2_O_2_. Therefore, they concluded that heat stress results in oxidative stress originating from the mitochondria and nuclear mutations.

Concerning yeast, caloric restriction (<2% of glucose) (Weinberger et al., 2007) and catalase inactivation (Mesquita et al., 2010) are approaches to extending its lifespan. These approaches elevate the concentration of H_2_O_2_ through the activation of superoxide dismutase, inhibiting the accumulation of ROS (Mesquita et al., 2010). The accumulating concentration of H_2_O_2_ is markedly below the threshold of toxicity. Furthermore, it could be shown that yeast cells in the stationary phase are more resistant to H_2_O_2_ than those in the exponential phase (Mesquita et al., 2010).

The third contributor to cellular stress are different epigenetic regulation pathways. Importantly, different yeast strains vary in their epigenetic stability. For example, *S. cerevisiae* have lost the ancient epigenetic pathways (e.g., methylation) and, thus, employ reduced epigenetic machinery compared with higher eukaryotic systems (Zemach et al., 2010). Another example of epigenetic instability is *S. pastorianus*, which is particularly unstable due to its chromosome copy number variation (Gorter De Vries et al., 2019).

As shown, yeast cell age can vary under different growth conditions.

Besides the above shown mechanisms of aging and age-related processes in yeast, relevant exogenous stressors as they appear in the industrial processing of yeast cultures will be discussed below.

## Aging Effects In Yeast Cultures Under Industrial Processing

Yeast cultures are widely used in industrial processes, e.g., in wine production or breweries. Within such processes, the used yeast cultures face multiple parallel factors that may affect cellular vitality, viability, and the maximal cell age. Because aging is a similar process that is common to all eukaryotes with highly conserved pathways, Frenk et al. hypothesized that the negative aspects of aging might be counterbalanced by making aged yeast cells more adaptable to new and stressful environments. In support of their hypothesis, the researchers were able to demonstrate that although younger yeast cells outcompeted older yeast cells of the same strain under optimal growth conditions in the metabolism of glucose, the older cells outcompeted the younger cells when alternative carbon sources, such as galactose, were solely available (Frenk et al., 2017). The underlying mechanism might be an increased stress response in the older cells that allows them to be more competitive in the presence of other environmental stressors.

While the influence of the environmental conditions and the yeast strains used on the formation of these aromatic substances has been investigated (Verstrepen et al., 2003), the significance of the yeast cell age on these components remains unknown. However, cellular factors and physiological functions have been shown to deteriorate with increasing cell age (Knorre et al., 2018). The consequences include a decrease in the synthesis and activity of ribosomes (Motizuki and Tsurugi, 1992; Reverter-Branchat et al., 2004), decreased growth and substrate uptake (Leupold et al., 2019) and the downregulation of genes encoding enzymes involved in glucose and energy metabolism. An example for such genes is *LAT1*, coding for components of the pyruvate dehydrogenase complex enzymes (Kamei et al., 2014). However, these enzymes are also essential for the synthesis of the three most important groups of aromatic compounds, namely higher alcohols, acetate, and ethyl esters, or the important precursor molecule acetyl-CoA for ester formation. However, what is missing in all these studies is how these fermentation by-products are influenced by cell age and the associated decrease in physiological function.

### Exogenous Stress

#### Sugar Stress

Yeast cultures are often used for the conversion of different kinds of carbohydrates through alcoholic fermentation. It is well-established that high concentrations of sugar in the surrounding liquid media may be a source of stress to the yeast cultures, since glucose is a possible inhibitor of cellular respiration (Crabtree effect) (Ibsen, 1961). Landolfo et al. (2008) showed that hypoxic alcoholic fermentation of high-sugar media was associated with increased levels of ROS. Thus, a resulting oxidative stress response negatively influenced the viability of the cell culture. Furthermore, they found that yeast cultures began to accumulate trehalose. Trehalose exerts protective effects on yeast cultures, which are mediated by two pathways. Firstly, it supports the integrity of the cell wall by binding to polar groups of phospholipids and substituting water in the cell wall. Secondly, it serves as a chaperone, thus preventing the aggregation of denatured proteins and stabilizing those in their native state. Accordingly, deficiencies in the synthesis of trehalose are associated with lower viability of fermenting cultures and lower production of ethanol (Trevisol et al., 2011). Furthermore, there is evidence that trehalose protects yeast cultures, particularly membranes, from lipid peroxidation during conditions of oxidative stress (Herdeiro et al., 2006).

Another important stressor in industrial processing is carbonyl stress resulting from the reaction of sugars, such as glucose or fructose with native proteins. It is suggested that this process results in advanced glycation end products. Studies in yeast have demonstrated that glycation inhibitors, like aminoguanidine, decrease the concentration of advanced glycation end products, extending chronological lifespan (Kazi et al., 2016). Similar data exist concerning carbonyl stress in yeast cultures mediated by fructose, resulting in higher levels of oxidative stress, α-dicarbonyl compounds, and carbonyl groups of proteins. It was proposed that these effects may explain the lower viability and reproductive capacity as well as higher cell mortality in fructose- vs. glucose-supplemented growth media (Semchyshyn et al., 2011).

#### pH Stress

Although yeast cultures show a broad tolerance for pH between 3.0 and 11.0 (Rogowska et al., 2018), viability and cell size are greatly influenced by the pH of the growth medium. Therefore, a differentiation between the inner pH in the yeast cell and impact of extracellular pH is necessary. The impact of the intracellular pH value due to proton pumps is explained later in this review. The impact of extracellular pH is explained below. The largest cell size and viability values were reported at pH 4.0 (Rogers et al., 2016; Salari and Salari, 2017). The best growth rate was determined to be at a pH of 5.0 (Aguilar-Uscanga and François, 2003). However, evidence suggests that these acidic conditions are not optimal for cell aging in terms of the chronological and replicative lifespan of yeast. Ongoing extracellular acidification of the culture medium reduces the replicative lifespan because acetic acid, an organic acid produced by fermentation, can cross the yeast plasma membrane resulting in acidification of the cytosol (Burtner et al., 2009) and a lower intracellular pH value. This effect can be compensated when the culture medium is buffered to a pH level of 6.0 (Burtner et al., 2009; Murakami et al., 2012) or by reducing the glucose concentration to 0.5%, leading to a decreased acetic acid formation and reduced medium acidification (Burtner et al., 2009). These data are consistent with the results obtained by extracellular acidification in other eukaryotic cell models, suggesting a common conserved eukaryotic stress pathway (Morgunova et al., 2017). The reasons responsible for the increase in chronological lifespan through dietary restriction or medium buffering are not elucidated. Caloric reduction results in extension of the replicative and chronological lifespan by changing molecular and cellular mechanisms (Leonov et al., 2017). Some of these mechanisms involve adjustments of the ethanol metabolism and growth rate (Tahara et al., 2013), trehalose metabolism (Kyryakov et al., 2012), mitochondrial morphology (Goldberg et al., 2009), and mitochondrial functionality. Moreover, they are linked to maintenance of ROS homeostasis (Goldberg et al., 2009; Ruetenik and Barrientos, 2015), cell cycle regulation (Leonov et al., 2017), reduction in ribosomal DNA (rDNA) recombination (Banerjee et al., 2020) and apoptotic markers due to fragmentation of the mitochondrial tubular network (Goldberg et al., 2009).

#### Salt Stress

There is also evidence that yeast cultures react differently to osmotic shifts in the exponential vs. the stationary growth phase. Cells in the exponential growth phase show a lower turgor pressure (0.05 MPa) and a greater relative cell volume decrease under hyperosmotic conditions than stationary phase cells (turgor pressure: 0.2 MPa) (de Marañon et al., 1996). However, the reported values of turgor for heterogenous yeast cultures ranged from 0.2 to 1.0 MPa (Proctor et al., 2012), and single-cell prediction suggested a turgor value of 0.2 MPa (Goldenbogen et al., 2016). Nevertheless, the direct measurement of cell turgor is currently not possible. Another research group showed that hyperosmotic growth conditions are associated with DNA strand breakage, a reduction in the number of cristae, mitochondrial swelling, chromatin condensation along with the nuclear envelope, and perturbances of the plasma membrane integrity. These effects appear to be mediated via metacaspase- and mitochondria-dependent apoptosis pathways (Silva et al., 2005). Finally, osmotic stress and ethanol stress have been reported to impact yeast cell morphology through a change in cell volume, resulting in yeast cell shrinkage and a rough surface (Pratt et al., 2003; Canetta et al., 2006).

#### Ethanol and Acetaldehyde Stress

To cope with alcohol and acetaldehyde stress, yeast cultures express several converting enzymes for detoxification. An important family of these enzymes comprises the aldehyde dehydrogenases with mitochondrial enzymes (*ALD4* and *ALD5*) and their cytosolic counterparts (*ALD2, ALD3*, and *ALD6*) (Dickinson, 1996; Meaden et al., 1997). It was shown that the regulation and activity of these detoxifying genes vary significantly between different growth conditions and strains, and may partially be induced by supplementation with ethanol and acetaldehyde in the growth medium (Aranda and del Olmo, 2003). Furthermore, it was found that different heat shock proteins (HSPs) (e.g., Hsp104p) are involved in the stress response to ethanol and acetaldehyde stress, suggesting a typical stress response to different extracellular stressors (Aranda et al., 2002).

Another essential molecule involved in stress tolerance against ethanol and acetaldehyde is the Batten disease protein 2 (Btn2p). This protein is involved in intracellular protein trafficking and, thus, the localization control of several intracellular proteins (Kama et al., 2007). Under acetaldehyde stress, the expression of *BTN2* is increased; however, it is reduced in the presence of ethanol. Surprisingly, the increased vulnerability to ethanol could be compensated by supplementation with arginine in the growth medium. It has been reported that *BTN2* deletion leads to increased vulnerability of yeast to higher concentrations of ethanol (Espinazo-Romeu et al., 2008). Thus, *BTN2* may be an important regulator of viability and vitality in yeast experiencing acetaldehyde or ethanol stress.

According to the literature, the consequences on yeast cell morphology and physiology are similar to those caused by hyperosmotic stress and ethanol stress, such as cell shrinkage and disrupted cell division (Gibson et al., 2007).

#### Oxidative Stress

As already mentioned, oxidative stress to yeast cells may occur endogenously and exogenously. Endogenous sources of RONS include the enzymatic activity of nicotinamide adenine dinucleotide phosphate oxidase (Salisbury and Bronas, 2015). Like mentioned in section Mechanisms of Aging, exogenous, non-oxidative stressors may trigger endogenous oxidative stress because, for example, high extracellular sugar concentrations may provoke an oxidative stress response (Landolfo et al., 2008; Semchyshyn et al., 2011).

As mentioned in Sugar Stress, strong connection exists between sugar and alcohol metabolism is evident in yeast. During the aging of yeast cultures, it was found that some proteins are oxidatively modified, thus reducing the *in-vivo* activity of these enzymes. A target of such oxidative damage is the alcohol dehydrogenase 1 gene promoter (Adh1p), catalyzing acetaldehyde conversion to ethanol (Reverter-Branchat et al., 2004). It was shown that an extra copy of the *adh1* gene is associated with prolonged survival in the stationary growth phase (as a surrogate marker of chronological lifespan) and a 30% extension of replicative lifespan determined by micromanipulation. It is assumed that Adh1p is oxidatively modified, and an extra copy results in a longer induction of antioxidant enzymes, such as superoxide dismutase and catalase (Reverter-Branchat et al., 2007).

Aging yeast cells also show noticeable signs of oxidative stress even without further external stressors. By comparing 5-day- and 3-month-old stationary yeast cultures, it was demonstrated that older cultures exhibit significantly lower glutathione levels, as well as superoxide dismutase and catalase activity. In line with lower antioxidative activity, the cultures showed increased protein oxidation and increased levels of protein carbonyl groups (Jakubowski et al., 2000). Regarding cultures consisting of chronologically aged mother cells and younger daughter cells, there is evidence that markers of oxidative stress are retained in mother cells during budding (Laun et al., 2001). Experimental data suggest that the cellular respiratory chain is a crucial regulator of endogenously-derived oxidative stress (Heeren et al., 2004; Drakulic et al., 2005). Notably, there is a strong connection between response to heat and oxidative stress (Davidson and Schiestl, 2001).

#### Heat Stress

In 1998, it was reported by Shama et al. that mild, non-lethal heat stress with two shocks of 2 h each extended the replicative lifespan of yeast cultures moderately from 19.4 to 21.3 budding events (*p* = 0.013) (Shama et al., 1998). In this case, the replicative lifespan was also determined by micromanipulation. The authors hypothesized that this effect may be conferred by the Hsp104p, which is assumed to be responsible for life extension due to transient heat stress. In previous work, it was shown that protein Hsp104p is also involved in yeast radiation stress responses (Boreham and Mitchel, 1994). Furthermore, it is established that Hsp104p is involved in replicative lifespan extension due to asymmetric cell division during budding. In this context, Hsp104p retains damaged proteins and cell structures in the mother cell (Higuchi-Sanabria et al., 2014) to rejuvenate the daughter cell.

Besides Hsp104p, there is also evidence that trehalose may be involved in the heat shock response of yeast, since the trehalose phosphate synthase genes (*TPS1* and *TPS2*) are activated by heat shock. In this analysis, a wild-type yeast strain was cultured for 72 h in sequence at 32, 34, 36, and 38°C. This culture resulted in a thermotolerant yeast strain (Kuroda and Ueda, 2018) reacting less sensitively to heat shocks due to the upregulation of stress-responsive genes, such as HSP-encoding and trehalose synthesis genes.

Within this context, trehalose may act as a protector (as mentioned earlier) to stabilize the native structure of proteins (Kuroda and Ueda, 2018) and prevent protein aggregation (Magalhães et al., 2018). After heat shock, trehalose is decomposed within ~1 h (Kuroda and Ueda, 2018), resulting in protein aggregation and assembly inside cells by interactions of unfolded and folded domains (Chiesa et al., 2020).

A more recent study conducted by Chernova et al. (2017) on heat shock reaction revealed a transgenerational memory for heat stress. This memory is mediated by the cytoskeleton protein Lsb2, which forms a metastable prion during heat stress, thereby triggering the conversion of other Lsb2 proteins into the prion form. In this context, prion formation is linked to the heat shock response, which contributed to maintaining protein homeostasis and protecting cells from the impacts of exposure to stress (Vabulas et al., 2010).

However, thus far, there is no empirical evidence regarding the potential influence of this heat stress memory on the resilience of yeast against new heat shocks or other types of stressors (Chernova et al., 2017).

#### Mechanical Stress

By pumping yeast cells into the fermentation vessel, as well as outside of the vessel at the end of the fermentation process, yeast cells were exposed to high mechanical stress. In addition, movements during the beer fermentation process, such as multiphase flow for the transport of nutrients, heat (Meironke and Böttcher, 2014), and agitation (Stoupis et al., 2003), suggest mechanical stress of the cells, influencing the process itself.

At present, there is limited knowledge regarding the impact of mechanical stress and shear stress on yeast cell aging. It has been shown that only the temperature and medium composition significantly affect the mechanical properties of the cell. Of note, stirrer speed or aeration rate do not affect the mechanical properties and cell wall strength of yeasts (Overbeck et al., 2015). Instead, mechanical shear stress has an impact on yeast cell morphology, metabolism, and viability. It is established that *S. cerevisiae* yeast is very tolerant to shear stress with a threshold of >1,292 Pa, resulting in non-significant loss of viability (Lange et al., 2001). Higher shear stress results in a significant increase in yeast cell death. Mechanical and hydrodynamic shear stress results in a decreased physiological state, reduced flocculation intensity, and increased levels of yeast extracellular proteinase A (PrA) (Stewart, 2018).

It was also revealed that cell-cell and cell-matrix contacts, mechanical forces, or environmental factors (e.g., changes in osmolarity, temperature, and pH) affect the function and activity of cells (Brewster et al., 1993; Dhanasekaran and Reddy, 1998; Chen and Thorner, 2007). Most of these processes lead to the modification of transcription factors in the cell nucleus. This process alters the gene expression pattern, ultimately initiating a reaction to the received signals.

Proteins of the extracellular signal-regulated kinase/mitogen-activated protein kinase (ERK/MAPK) pathway play an important role in signal transduction. These pathways are activated by stimuli in the form of the aforementioned environmental factors via membrane-associated receptors and regulate different cellular processes, such as growth, differentiation, apoptosis, and stress regulation (Guo et al., 2020).

In most organisms, the MAPK signal cascade is comprised of three essential enzymes (Guo et al., 2020) that are connected to a G protein. These enzymes activate the following in sequence: (I) a serine/threonine-specific MAPK kinase kinase (MAPKKK) (membrane shuttle kinase), which phosphorylates and activates (II) a MAPK kinase (MAPKK) (dual-specificity kinase), which in turn activates (III) a specific serine/threonine MAPK (nuclear shuttle kinase) through serine/threonine and tyrosine phosphorylations (Dhanasekaran and Reddy, 1998; Kolch, 2000; Cargnello and Roux, 2011). This MAPK usually translocates directly into the cell nucleus to modify transcription factors or other target proteins.

This pathway is activated by high osmolality in the form of the protein kinase high osmolarity glycerol (*HOG1*), which triggers the activation of the MAPK pathway in sequence (Brewster et al., 1993). In contrast, the chemical and physical stress of the cell wall results in activation of the cell wall integrity pathway, which activates a MAPK module (Lee et al., 2020). The only difference in these two activation cascades is the initiating step of the G protein-coupled receptor. Therefore, the signal cascades of mechanical stress and osmotic pressure appear markedly similar.

### Endogenous Stress

#### Genomic and Epigenomic Stability

The effects of aging on yeast cells can be determined through changes in cell wall composition. Furthermore, changes in membrane fluidity are also evident in aging yeast cells in addition to extrachromosomal rDNA circles, aneuploidy, and nucleolus organization, which function as surrogate markers for the genomic and epigenomic stability of yeast cells.

Aneuploidy and nucleolus disorganization are associated with decreases in the chronological lifespan of yeast. Yeasts have numerous molecular pathways to stabilize the DNA content and maintain a stable genome allowing mutation tolerance (Skoneczna et al., 2015). However, these systems are not fail-safe. In a study investigating the targeted loss-of-function mutations of several cell cycle checkpoint control genes (e.g., spindle assembly checkpoint *MAD1*, serine/threonine-protein kinase *BUB1* and *TEL1*, and mitotic checkpoint *BUB2*), the impact of these mutations was examined in the context of changes in the structure and function of the nucleolus that is mediated by aging. Nucleolus fragmentation and differences in size, as well as in the size ratio of the nucleus to the nucleolus, were associated with aging in the affected yeast strains. Additionally, these changes indicated increased levels of oxidative stress, DNA damage, and aneuploidy (i.e., chromosomal number aberrations rather than translocations and other structural chromosomal damage). All mutated yeast strains showed a decreased chronological lifespan, with the *BUB1* mutant being the most robust effect inducing both aneuploidy and a decrease in lifespan. Researchers also reported that aging increased the nucleolus size, which may have caused the observed aging-related nucleolus fragmentation. In contrast, aging-related oxidative stress may cause chromosome XII instability, leading to rDNA-instability and aneuploidy events (Lewinska et al., 2014).

Extrachromosomal rDNA circles also determine the replicative lifespan of yeast cells, since they arise from the 150 ± 50 copies of rDNA and are found on chromosome XII in yeast (Sinclair and Guarente, 1997). As a result of recombination, copies are “cut out” and appear as plasmids in the cell nucleus. Since each copy also contains an origin of replication (Clyne and Kelly, 1997) [“autonomous replicating sequence” (Sinclair and Guarente, 1997)], these circles can reproduce independently of the chromosomes (“self-replicating”). Such “plasmids” preferentially remain in the mother cell during mitosis since older yeast cells contain a higher number of circles and show a reduced lifespan compared with younger cells (Falcón and Aris, 2003).

Furthermore, extrachromosomal ribosomal DNA circles accumulate with age. However, other high-copy protein-coding circular DNAs accumulate during yeast cell aging, indicating that the environment influences yeast cell genetics (Hull et al., 2019). This extrachromosomal circular DNA accumulation may serve as a pool of heterogeneous genetic material, allowing rapid adaptation of aged cells to environmental changes (Hull and Houseley, 2020).

In this context, researchers further assumed that the replication of extrachromosomal rDNA circles contributes to nucleolar fragmentation and plays an essential role in limiting the lifespan of yeast cells (Sinclair and Guarente, 1997; Johnson et al., 1998; Lewinska et al., 2014). According to Kobayashi and Sasaki (Kobayashi and Sasaki, 2017), the regulation of rDNA stability and integrity is a complex process as observed from a sample of 4,800 budding yeast strains after examining 700 diverse types of genes. Additionally, ~50 genes highly involved in regulating the rDNA copy numbers were identified, and some mutants showed abnormalities in other chromosomes than chromosome XII due to unstable rDNA (Kobayashi and Sasaki, 2017). Therefore, it can be concluded that ERCs are not the only factor affecting aging. Instead, rDNA instability also affects aging in yeast to a great extent. Perhaps rDNA instability has a much higher impact than ERCs, especially in higher organisms (Lee and Ong, 2021).

Besides rDNA stability and copy number, telomere length has an impact on yeast cell morphology. Telomere senescence resulted in an increase in the diameters of unbudded cells from 5.2 to 8.2 μm and of budded cells from 6.3 to 10.1 μm, indicating an increase of 58 and 60%, respectively (Ghanem et al., 2019). This change in cell diameter negatively influences the optimal ratio of DNA:cytoplasm, supporting the observed non-optimal cell function (Neurohr et al., 2019). Additionally, a shift in flocculation behavior and the loss of growth capability could be determined (Ghanem et al., 2019) due to telomere-induced cellular senescence.

#### Mitochondrial Dysfunction

Mitochondrial aging, which is characterized by decreased mitochondrial DNA content and extensive oxidative damage in mitochondrial proteins, is associated with chronological aging both in mammals and yeast cells. Mitochondrial aging can act as an independent stress factor for yeast in the commercial brewing process, underscoring the importance of the involved mechanism (Powell et al., 2000a). Mitochondria are relatively independent of the cellular mechanism; they replicate independently within the cells and contain independent repair mechanisms (RAD-52 related recombinase system) to repair DNA double-strand breaks (Chen, 2013). Nevertheless, some nuclear genes regulate the mitochondrial DNA content (Zhang and Singh, 2014). Aging cells accumulate progressive alterations to their biosynthetic and oxidative metabolism. These aging processes are conserved between yeast and mammalian cells and contribute to the increasing metabolic dysfunction of aging cells (Baccolo et al., 2018).

Oxidative stress in mitochondria results in overproduction of ROS. These ROS induce DNA mutations and damage the mitochondrial respiratory chain (Guo et al., 2013). Damaged mitochondria result in the accumulation of ROS, which is also associated with cellular dysfunction and reduced replicative lifespan by disrupting protein homeostasis in yeast (Yi et al., 2018). Leadsham et al. showed that mitochondrial dysfunction results in accumulation of ROS and suppresses the endoplasmic reticulum-associated degradation. This degradation leads to higher ROS production from the endoplasmic reticulum due to the suppression of the antioxidant defense and endoplasmic reticulum-resident nicotinamide adenine dinucleotide phosphate oxidase (Leadsham et al., 2013). This upregulated superoxide production results in the accumulation of a higher concentration of H_2_O_2_, thereby leading to loss of redox homeostasis. Furthermore, respiratory-deficient cells exhibit reduced RLS due to the intracellular ROS and higher amount of oxidatively damaged proteins, resulting from mitochondrial dysfunction (Yi et al., 2018).

#### Oxidative Damage to Proteins and Protein Aggregates

The levels of ROS in yeast influence the mitochondrial function and protein homeostasis. Besides, these ROS lead to oxidative modifications of proteins, affecting their function and conformation. Oxidative modifications can change the protein function by either directly reducing/inhibiting the function or changing the conformation of the protein (Kikis et al., 2010). These changes in activity and conformation decrease protein stability. Oxidized and misfolded proteins tend to form protein aggregates, which are potentially cytotoxic for the cell and directly associated with accelerated aging (Grune et al., 2004; Hartl et al., 2011). A recent study showed that with increasing age, damaged proteins are increasingly retained, and this process is directly linked to the asymmetric division of the yeast cell (Borgqvist et al., 2020).

### Exogenous and Endogenous Stress on Yeast Cells

**Figure 3:**
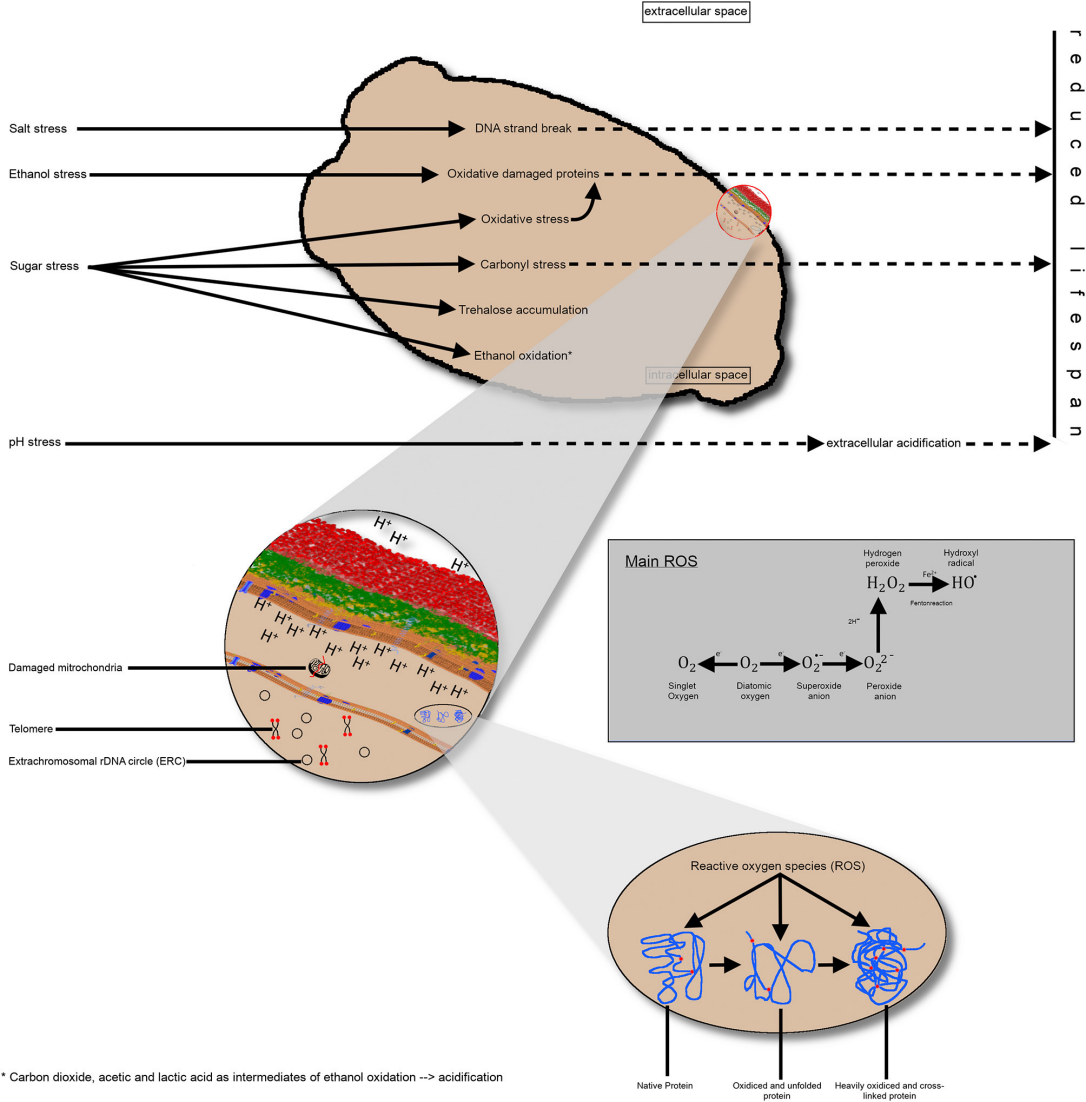
Schematic representation of the impact of different industrial stress conditions on the yeast cell. **(A)** Singular stress conditions altering the metabolic pathways and resulting in consequences, such as DNA strand breakage, protein oxidation, carbonyl stress, or ethanol oxidation, thereby producing different intermediates. Collectively, these effects reduce the replicative lifespan of yeast cells. **(B)** A more detailed view of the intracellular processes and endogenous stress, such as damaged mitochondria or ROS impact on proteins. Additionally, the telomere length until a critical length and the accumulation of extrachromosomal, rDNA circles reduce the replicative lifespan. Impairment of proton transfer from the inner to the extracellular space results in intracellular acidification, non-optimal enzyme activity and, thus, a shorter lifespan. **(C)** Visualization of protein oxidation; left: native protein; middle: unfolded protein due to oxidative effects; right: heavily oxidized protein without further function. rDNA, ribosomal DNA; ROS, reactive oxygen species.

As discussed earlier in this review, there is limited knowledge regarding the cumulative effect of multiple stressing influences during the industrial processing of yeast. Evidence exists that different stressors trigger metabolic pathways associated with intracellular acidification or an increase of ROS resulting in damaged proteins and mitochondria (Figure 3). Hence, it can be assumed that the stressors exert an influence on the chronological and reproductive age of yeast cells, as well as their physiological state. Factors influencing this physiological state are the intracellular pH value due to an impaired proton transfer across the cell membrane, the oxidative effect on proteins leading to lower enzyme activity, or damaged mitochondria resulting in more ROS oxidative effects in the yeast cell.

There is evidence that cell cycle duration, cell volume, and the number of budding events per time unit differs significantly under different stress conditions. In industrial processes, yeast environmental conditions can be extremely harsh in particular, through the accumulation of desired metabolites, such as ethanol, causing premature aging. However, it is also possible that yeast strains adapt to stressors by the increased stress response, and aneuploidy might be a factor in the upregulation of stress responses. A study evaluating yeast in a long-term culture with sublethal ethanol concentrations showed that yeast cells indeed accumulate age-related changes. However, throughout 100 generations, yeast cells showed gains in numbers of chromosomes I, III, and VI and increase in copy numbers in genes that were involved in stress responses and metabolic processes. Moreover, through the action of ROS, sub-cytotoxic ethanol concentrations increased growth rates in yeast and increased the expression of several sirtuin proteins, namely Sir1p, Sir2p, and Sir3p, as well as transcription factor Rap1p (Adamczyk et al., 2016).

Sirtuins form a family of conserved protein deacetylases that are NAD^+^-dependent. Sir2 was the first characterized sirtuin and functions as a histone deacetylase, enabling the silencing of heterochromatin. This function prolongs by an incompletely understood mechanism in the replicative life span of yeast. Its deletions accelerate replicative aging, whereas increased gene dosage inhibits replicative aging (Wierman and Smith, 2014).

These results suggest that adaptive stress response can include acquiring additional chromosomes and genes if they are coding for the protein necessary to mitigate the stress. Nevertheless, similar to mammalian cells, in yeast cells, there appears to be an optimal cell size, DNA content, and cell size/DNA content ratio for optimal life span, which might differ depending on environmental conditions (Veitia, 2019). Furthermore, the retention of damaged protein in the mother cell has a high impact on the maximum replicative lifespan of yeast cells (Borgqvist et al., 2020).

Some further stressors that are tightly linked to the aging process are ROS and RNS, which in yeast can be shown to accelerate senescence, similar to what occurs in other eukaryotic cells when yeast is grown under aerobic conditions. The ability of yeast to grow under anaerobic conditions using fermentation only allows it to grow in an environment with low-to-negligible ROS concentrations, enabling researchers to examine the influence of other environmental stressors on the aging process (Eleutherio et al., 2018). In addition, the industrial process produces several intense environmental stressors that influence the aging process of yeast, although they are still incompletely understood.

However, as shown in the sections above, most research has been performed to determine the replicative age and stress reactions to singular stress conditions. In industrial processes, the yeast cell is confronted with multiple parallel stress factors affecting its metabolic pathways and physiological state. Thus, there is a lack of evidence on the interaction of multiple stress conditions and their impact on yeast cells' replicative aging and lifespan.

## Impact Of Aging On Yeast Cells

**Figure 4:**
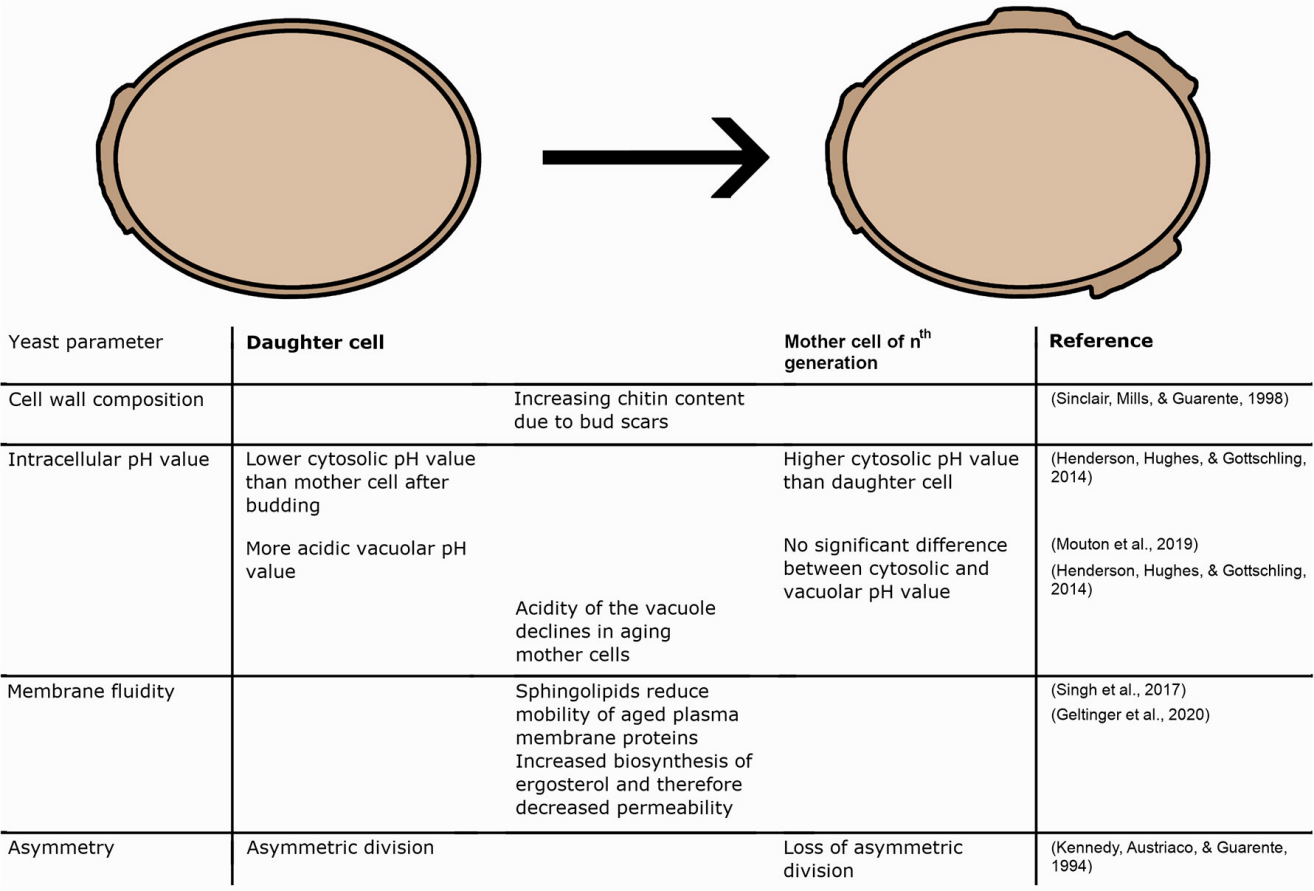
Illustration of different intracellular changes in daughter cells, mother cells, and changes during the aging process.

As mentioned above, yeast cells and cultures are utilized as models for the investigation of the aging processes. Against this background, several aspects appear as significant changes when aging yeast cells are examined (Figure 4). Firstly, some alterations can be found in the wall composition of yeast cells. This aspect appears particularly important concerning the replicative aging of yeast. Secondly, the intracellular pH value will be discussed as a surrogate marker for yeast vitality and longevity. As the last aspect of aging processes in yeast cells, alterations in membrane fluidity can be found in aging yeast cells and are mainly involved in ethanol tolerance and replicative events.

### Alterations in Cell Wall Composition

Alterations in replicative aging in yeast are influenced by environmental and genetic factors, resulting in different protein impairments and slight changes in the composition of the cell wall.

Concerning cell wall composition, Bulawa and Osmond (1990) assumed that the septum contains large amounts of polysaccharide chitin, resulting from the replicative capacities of yeast cells. Initially, it was thought that chitin was synthesized by chitin synthase; however, cloning experiments proved that chitin synthase 1 (*CHS1*) was not essential for chitin synthesis.

Cabib et al. (1989) showed that lysis of *CHS1*-deficient cells occurred at the end of the cell cycle, as a result of a cell wall lesion in the center of the birth scar. Of note, membranous material appeared to escape from this hole. This lesion was not found in wild-type strains.

**Figure 5:**
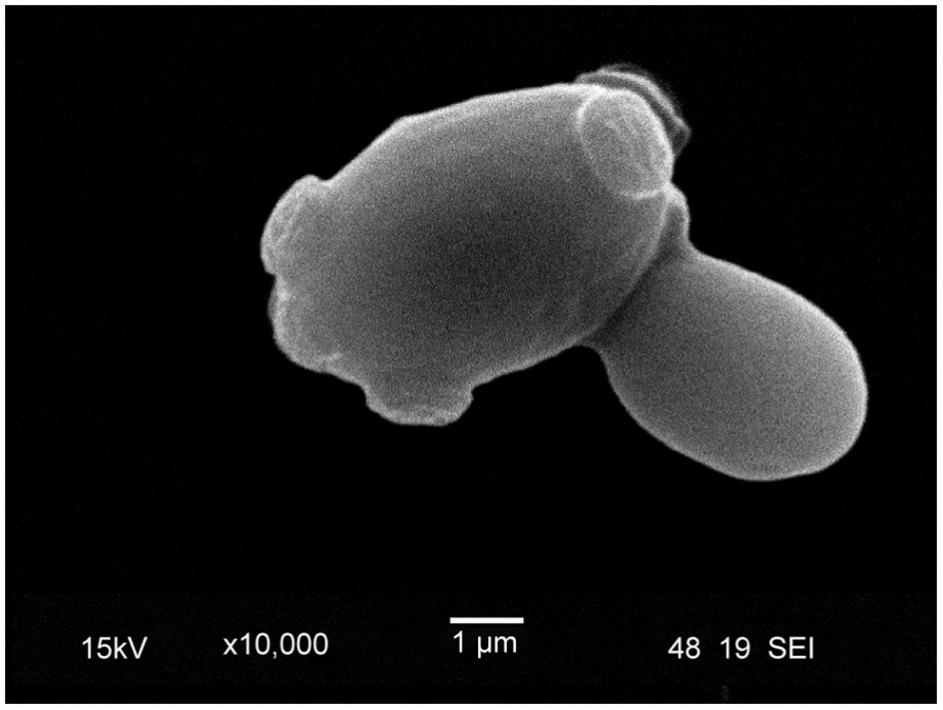
Scanning electron microscope image of lager yeast (TUM 34/70) with bud scars. During the budding process, a daughter cell is created, and the surface, on which the bud formed, is covered by a chitin ring, a marker of replicative aging. The inner surface of the chitin ring consists of beta-glucan.

Subsequently, it was shown that the total amount of chitin in yeast septa is low; nevertheless, chitin is crucial for cell viability (Cid et al., 1995; Cabib et al., 2001). Furthermore, there were reports that yeast cells lacking *CHS1* and *CHS2* show average growth, chitin content, and cell division, which led to the discovery of *CHS3* (Bulawa and Osmond, 1990). Emerging evidence suggested, in the following years, that chitin synthases may play a crucial role in the replication and viability of yeast cells. Specifically, at bud emergence, a chitin ring is formed at the neck of the cell (Figure 5) through a *CHS3*-dependent process (Shaw et al., 1991). Nevertheless, the formation of the primary and secondary septum requires Chs2p activity rather than Chs3p activity (Cabib et al., 1996, 2001). Further investigations by Cabib and Schmidt (2003) aimed at elucidating the role of *CHS3* in *CHS2*-deficient cells. Using cell cycle synchronization with inhibition of *CHS3* by nikkomycin Z in *CHS2*-deficient cells, they showed that only chitin synthesis during septa formation is essential for the viability of yeast cells. They concluded that construction of the chitin ring is crucial for the integrity of the mother-bud neck (Cabib and Schmidt, 2003).

These results imply an involvement of the cell wall and septa composition in the viability and replicative capacities of yeast cells. However, there is growing evidence that specific cell wall properties (e.g., cell wall composition, surface wrinkling or thickness) may be age-related (Egilmez et al., 1990; Cabib et al., 1997; Powell et al., 2000b). Based on this evidence, Molon et al. (2018) investigated the influence of different genes associated with cell wall formation and integrity maintenance on the replicative aging of yeast as the number of reproduction events against the reference strain BY4741 used as control. They tested five gene deletions concerning the impact of the encoded proteins on cell wall composition: (I) *chs3*Δ; (II) chitinase 1 (*cts1*Δ); (III) 1,3-β-glucan synthase (*fks1*Δ); (IV) transcriptional regulator of cell wall chitin and glucan synthesis *(knr4*Δ) and; (V) protein for the synthesis of α-1,6-mannan (*mnn9*Δ).

Molon et al. suggested that the impairment of genes involved in cell wall protein mannosylation (*mnn9*Δ) and chitin synthesis (*chs3*Δ) decreased budding lifespan, whereas impairment of knr4 significantly increased lifespan (Molon et al., 2018).

Eigenfeld et al. showed that industrial stress conditions have an impact on the morphology of yeast cells. Scanning electron microscope images have indicated a change in cell morphology from the smooth surface of pure yeast to a rough deformed surface with indentations of repitched yeast cells (Eigenfeld et al., 2020).

Furthermore, protein impairment and morphological changes are not the only impacts of stress on cell wall composition and morphology. Moreover, it was shown that the overall cell wall composition varies significantly in terms of β-1,3-glucan, 1-6-glucan, mannan, and chitin content depending on different culture parameters, such as carbon source, pH, temperature, aeration, and nitrogen limitation (Aguilar-Uscanga and François, 2003). Based on these results, it can be concluded that environmental parameters and stress conditions result in impairment of: (I) proteins for mannosylation and chitin synthesis and (II) regulation factors for chitin and glucan synthesis. This impairment results in an increase or decrease in replicative lifespan.

### Yeast Vitality and Intracellular pH

Plasma membrane ATPase, which regulates the intracellular pH, is crucial for the growth of yeast cells (Serrano et al., 1986). This could be because the uptake of molecules, e.g., amino acids (Eddy and Nowacki, 1971; Seaston et al., 1973; Cockburn et al., 1975) and maltose (Serrano, 1977), are interconnected with the uptake of H^+^. The first experiment regarding intracellular pH and viability was conducted in 1995 by Imai and Ohno (1995). They found a strong positive correlation between the viability of brewer's yeast and intracellular pH when a low external pH was applied (*r* = 0.960; *p* = 0.001). The same correlation was also observed for *S. cerevisiae* yeast (*r* = 0.950; *p* = 0.001). Thus, it can be concluded that a high intracellular pH is a pivotal surrogate marker of the vitality of yeast. On the other hand, it could be assumed that intracellular acidification is associated with cellular senescence and lysis of yeast cells due to a non-optimal environment for intracellular enzyme activity. Therefore, a low intracellular pH is associated with low yeast vitality.

Indeed, it was found that amiodarone, a potent inducer of apoptosis in yeast, exerts its effects through the mitochondrial protein Ysp2. This protein is involved in cellular apoptosis caused by intracellular acidification following excessive ROS production and death (Sokolov et al., 2006). Furthermore, it was shown that Na^+^ (K^+^)/H^+^ exchanger Nhx1p is crucial for intracellular pH to control vesicle trafficking and associated metabolic processes. *NHX1*-deficient yeast cells are more vulnerable against external low pH stress, showing signs of intracellular acidification and trafficking defects (Brett et al., 2005) and, thus, younger cell age due to earlier acidification.

Furthermore, an interconnection between the age and metabolic activity of yeast cells was revealed. Aged mother cells are more resistant to osmotic stress and have higher vitality than old daughter cells, as measured by an increased respiration rate (Svenkrtova et al., 2016). The respiration rate and sugar consumption were also used to measure vitality in a study showing that loss-of-function mutation of thioredoxins leads to reduced vitality of yeast cells. This underscores the importance of redox-systems, which are often affected by aging processes, for yeast vitality (Picazo et al., 2019). Although environmental acetic acid and ethanol concentrations decrease yeast vitality, some genes involved in the aging process can also reduce the production of acetic acid and increase ethanol tolerance (Orozco et al., 2013).

### Alternations in Membrane Fluidity

The composition of different membranes markedly influences membrane fluidity. In yeast, sterols are prominent compounds found in numerous plasma membranes and determine membrane fluidity. Of note, they are also involved in protein sorting, vesicle formation, cytoskeleton organization, endocytosis, or replicative events (Heese-Peck et al., 2002; Tiedje et al., 2007; Aguilar et al., 2010; Jacquier and Schneiter, 2012; Caspeta et al., 2014). Kodedová and Sychrová (2015) examined the effects of disruption of different genes involved in the biosynthesis of ergosterol on membrane potential, salt tolerance, and other vital characteristics of yeast cells. It was shown that disruption of erg4 and *erg6* exerted a crucial effect on the integrity of the plasma membrane, indicating strong hyperpolarization. The relative membrane potential of the *erg5*-depleted mutant showed a similar membrane potential to that of wild-type cells.

Furthermore, the strains showed different responses to osmotic and pH stressors (0.8 and 1.2 M NaCl; 2.5 M glucose, 2.8 M sorbitol, and pH 3.0). In all cases, the wild-type strain BY4741 and the erg5-deficient strain showed the highest tolerance, while the other strains exhibited a significantly impaired tolerance against these stressors (Kodedová and Sychrová, 2015). On the other hand, there is evidence that membrane fluidity and yeast cell viability are positively correlated based on the ethanol tolerance of yeast cells. There is evidence that low membrane fluidity is correlated with higher ethanol tolerance and, thus, an increased viability (Ishmayana et al., 2017).

## Methods For Assessing Aging-Related Population Dynamics

Although several mechanisms of yeast aging are known, techniques for the precise assessment and quantification of the proportion of aging cells in a yeast population are essential. Such methods are important for industrial applications that rely on defined metabolic and genomic processes of microorganisms that differ in the singular cell age. As shown in section Aging Effects in Yeast Cultures Under Industrial Processing, stress conditions have an impact on the maximum cell age. Furthermore, the available sugars influence the age distribution, resulting in varying amounts of aroma compounds. Therefore, a detailed evaluation of the quantitative age distribution in yeast populations is necessary for deep understanding of the interconnection between singular cell age and the course of the fermentation process. The techniques used to quantify the maximal bud scar number of yeast and novel technological approaches for age determination are presented in the following sections.

### Micromanipulation Techniques

Since there is no precise mechanism for estimating the age of higher species, aging is measured as a cumulative result of various physiological processes and changes that involve damage and repair events. Early experiments to estimate the lifespan of yeast cells by micromanipulation and counting of budding events in mother cells revealed that mother cells could be distinguished from daughter cells based on scars on their surface (Mortimer and Johnston, 1959). According to this technique, the aging process of the mother cell is studied after separation from daughter cells during a budding event throughout a microscopic single-cell observation process. The mean lifespan was 23.9 ± 8.3 generations, indicating that yeast cells are mortal.

However, classical micromanipulation is unsuitable for determining the age distribution in yeast due to the time involved in the process and focus on total lifespan of single yeast cells (Liu et al., 2015; Hohnadel et al., 2018).

### Centrifugal Elutriator

A fundamental method for the enrichment of older yeast cells, the centrifugal elutriator, was published in 1995 (Woldringh et al., 1995). The machine is filled with a defined culture of yeast cells, which are separated by centrifugal forces based on their size (daughter cell: 2–4 μm, mother cell: 6 μm) (Svenkrtova et al., 2016). New daughter cells are thereby washed out of the chamber, and the system yields older or senescent yeast cells. Asymmetric cell separation is associated with a size discrepancy between the daughter and mother cell, resulting in a difference in sedimentation coefficient and cell size (Marbouty et al., 2014). The advantage of using this method is that a high number of daughter cells can be separated and also a continuous separation of daughter cells is possible.

Disadvantages of the method are (I) the dilution factor (Figdor et al., 1984), (II) the size-dependent fractionation (mother cells vary in their absolute size) (Kukhtevich et al., 2020) and (III) the varying size of the total population due to exogenous factors e.g., nutrient availability (Leitao and Kellogg, 2017). Therefore, fractionation using centrifugal elutriation may not provide robust results (Svenkrtova et al., 2016).

### Physical Immobilization, Mother Enrichment Program and Microfluidic Devices

Physical immobilization by tagging mother cells with biotin and immobilizing them using streptavidin-covered magnetic beads or streptavidin-covered columns is used in another approach (Denoth Lippuner et al., 2014). After separation, purification of >99% of old cells was determined and used for the subsequent analysis of telomeres in old cells (Smeal et al., 1996) or yeast age control in batch or continuous fermentation (Kurec et al., 2009).

Recently, genetic systems have been developed to separate younger from older yeast cells. The Mother Enrichment Program is based on the expression of a beta-estradiol induction Cre recombinase, which occurs only in daughter cells (Lindstrom and Gottschling, 2009). Two essential genes, namely ubiquitin carrier protein 9 (*UBC9*) (encoding the SUMO-conjugating enzyme) and cell division control protein 20 (*CDC20*) (encoding an activator of the anaphase-promoting complex), were disrupted. This process led to a *LOX*-mediated selection against daughter cells due to the growth of both mother and daughter cells in the absence of the inducer. In contrast, only mother cells will continue to divide after induction, whereas the daughter cells will be arrested at the M-phase. This kind of mother-daughter separation uses genetic modified systems for analyzing the genetic instability on chromosome XII (Lindstrom et al., 2011) or age-related epigenetic changes (Feser et al., 2010).

However, there are also microfluidic dissection platforms, trapping mother cells under soft elastomers resulting in the microscopic monitoring of yeast cells throughout the whole lifespan (Lee et al., 2012). This method enables the monitoring of 50 cells simultaneously. Another approach is the use of novel microfluidic device retaining mother cells in the chamber and flushing away daughter cells, allowing a single-cell observation (Xie et al., 2012). Through such systems, which mostly utilize microfluidic devices, it is possible to examine the morphologies of cells and organelles, cell cycle dynamics, and various molecular markers and track individual cells over the whole lifespan, which is typically up to 3 days (Lee et al., 2012; Xie et al., 2012). The limitation of this approach in yeast age measurements is the lack of high-throughput measurements of replicative aging in representative cell numbers as well as age distributions. Furthermore, this method is limited due to the size difference between mother and daughter cells. Instead, these methods focus on single-cell events and the tracking of individual cells throughout their lifespan.

A novel approach for the high-throughput analysis of replicative aging is a microfluidic platform known as high-throughput yeast aging analysis chip (HYAA-Chip) (Jo et al., 2015). Using this method, a maximum of 8000 individual yeast cells can be trapped in cup-shaped structures and retained for replicative lifespan analysis. Compared with the abovementioned methods, the system works independent of the cell size and works with high trapping efficiencies. However, quantitative analysis of age distribution is possible neither using the classical microdissection nor using the previously published microfluidic designs because information regarding the exact replicative age of the initial mother cells and composition of mother and daughter cells is unknown.

Previous analyses have always involved constant singular stress conditions to understand the aging of yeast cells. However, studies of the effects of multiple parallel stress conditions, which can also interact and influence replicative growth, have not been performed.

### Fluorescence Coupled Flow Cytometric Methods

Flow cytometric methods have been employed to determine the population dynamics of yeast cultures. During this process, different dyes can be bound to specific sub-populations and quantified by their fluorescence signals at a single cell stage (Davey and Winson, 2003; Calvert et al., 2008; Ambriz-Aviña et al., 2014). Of advantage is the possibility of high-throughput screening through real-time analysis techniques by using a range of physiological markers, such as the yeast cell cycle, viability and vitality. With this method, it is generally possible to get insight into the propagation and morphological characteristics of budding yeast cells (Lee et al., 2012; Xie et al., 2012). Here, it was possible to examine various gene expression patterns under different growth conditions, and yeast viability is assessed after differentiating between viable and dead cells.

Therefore, the advantage of flow cytometry is the fast measurement of statistically significant numbers of cells, as well as the simultaneous measurement of more than one physiological, metabolic or morphological parameter. Still, there is the limitation regarding the availability of fluorescence dyes in specific wavelength, as well as the fact that most fluorescence dyes were irreversible and invasive.

Kurec et al. (2009) used bud scar staining for the first time in high-throughput (analysis of 5,000 cells) combined with flow cytometry. Two fractions of yeast cells were generated using biotinylation. Bud scars were irreversible stained using Alexa Fluor 488-coupled wheat-germ agglutinin, followed by the subsequent measurement of fluorescence intensity. The number of bud scars of 50 cells was microscopically counted and correlated with the average fluorescence intensity, indicating a linear relationship, which allows a mean cell age estimation. Furthermore, by use of the average fluorescence intensity and the linear relationship, the calculated absolute number of bud scars could be used to estimate the time point of replacing the aged biocatalyst during long-term continuous beer fermentations (Kurec et al., 2009). Still there is room for improvement, because as described in section Aging in Yeast the distribution of each age fraction are non-normal distributed, so average intensities seem prone to errors. Furthermore, yeast cells show an autofluorescence (Surre et al., 2018) which was not taken into account in the calculations of fluorescence intensities.

To date to the best of our knowledge, a non-invasive approach for quantitative yeast cell age determination in heterogeneous cultures has not been conducted using high-throughput methods. The development of novel methods is necessary to overcome the challenges in analyzing and determining the exact age of yeast cells. Counting of the budding scar formations can serve as a useful tool to understand age-related transformations in yeast cells as these scar formations are considered a potential marker for analyzing the replicative aging process.

To sum it up, all methods presented in chapter 5 have one or more of the following disadvantages in each, thus limiting a holistic and reliable age fractionation. These are the (I) lack of high-throughput capacity, (II) not selective separation of heterogeneous yeast cells in more than two age fractions, and (III) use of genetic modified organisms for separation instead of wild-type strains, used in industrial processes of beverages. Furthermore, (IV) the cost and availability of suitable dyes or fluorochromes linked to antibodies present a practical limitation (Sendid et al., 2008).

Therefore, a new approach for fluorescence coupled flow cytometry could fill the gap of high-throughput, non-invasive determination of yeast cell age. A possible future approach comprising the advantages and minimizing the disadvantages shown could be the use of binding domains combined with fluorescent proteins for bud scar staining of yeast cells due to the non-invasiveness of binding proteins, as well as the possibility of protein modification. The resulting stained yeast cells can (I) be measured in flow cytometry for high-throughput measurements, resulting in real time analysis of significant numbers of yeast cells and their corresponding single cell fluorescence data. Furthermore, (II) the non-invasive reversible attached protein linker could serve as tool for fractionation in more than daughter and mother fractions by use of protein specific interactions. Especially yeast cells bound with a binding domain-containing protein can serve as a basis for the fluidic manipulation of yeast cells by age clusters. This manipulation results in different-aged cells by replicative age, which can serve as calibration standards in flow cytometry. Therefore, the fluorescence intensities of defined aged cells were used for the quantitative analysis of cell populations instead of the average numbers of bud scars, resulting in an improvement. Additionally, (III) no genetic modification of yeast cells for separation is necessary, so yeast samples from industrial processes could be directly analyzed and fractionated yeasts can be used for further applications. (IV) The protein linker could be expressed in a recombinant way, so there is no limitation in its availability, as well as there is a possibility of further optimization by using fluorophore wavelength with low autofluorescence. To summarize, a designed protein for the visualization of bud scars of yeast cells has huge potential in industrial fermentation processes due to versatility. Using the described new approach, a detailed quantitative analysis of cellular age distributions in yeast populations would be possible, as well as new separation techniques.

## Conclusions

According to the currently available literature, the aging process of yeast cells depends on epigenomic integrity, mitochondrial dysfunction, and damaged proteins due to stressors, such as sugar, salt, pH value, ethanol, heat, and mechanics. There are currently few approaches for the high-throughput analysis of the impact of these stress factors on replicative aging in complex media. At present, there is limited research concerning the cumulative effect of multiple stressors in yeast cells during industrial processes. Nevertheless, research studies have illustrated the influence of different stressors, such as oxidative stress, pH, and heat on the metabolic pathways of yeast cells upon an increase in the intracellular levels of RONS. Therefore, it can be asserted that individual stressors exert significant effects on the reproductive and chronological aging of yeast cultures, mainly affecting the duration of the cell cycle, cell size, and budding events. Compared with established procedures, which are either time consuming, less sensitive, invasive, use genetically modified organisms, or focus only on daughter cell fractions, the use of fluorescence coupled flow cytometry as a high-throughput method could accurately estimate the aging of yeast cultures in industrial processes. This approach would assist in analyzing the impact of stress conditions on age-related population dynamics. The usefulness of this method is based on the availability of a calibration standard for yeast cell age through, for example, fractionation of yeast cell populations by singular cell age.

## Author Contributions

ME and RK planned and designed the manuscript. ME collected and reviewed the literature, and wrote the manuscript. ME, RK, and TB discussed the manuscript. RK and TB edited the manuscript. All authors have given approval to the final version of the manuscript.

## Conflict of Interest

The authors declare that the research was conducted in the absence of any commercial or financial relationships that could be construed as a potential conflict of interest.
